# ‘Amazing and Daunting’: Providers’ Perspectives on the Implementation of Neurofeedback

**DOI:** 10.21203/rs.3.rs-6719627/v1

**Published:** 2026-03-24

**Authors:** Whitney K Norris, M. Kathryn Allison, Ellen Shaw-Smith, George Pro, Geoffrey Curran, Martha Rojo

**Affiliations:** University of Arkansas for Medical Sciences; University of Arkansas for Medical Sciences; University of Arkansas for Medical Sciences; University of Arkansas for Medical Sciences; University of Arkansas for Medical Sciences; University of Arkansas for Medical Sciences

**Keywords:** neurofeedback, EEG biofeedback, implementation science, Consolidated Framework for Implementation Research (CFIR)

## Abstract

Neurofeedback has been used to help treat a wide variety of mental health issues. Despite studies documenting its clinical effectiveness, there is a significant gap in the literature on the determinants of neurofeedback’s use in mental healthcare practice. This study sought to fill this gap by utilizing the Consolidated Framework for Implementation Research (CFIR) to explore providers’ (n=20) perspectives on the implementation of neurofeedback via semi-structured one-to-one interviews. The qualitative analysis utilized grounded theory techniques to create an inductive codebook to reflect the implementation process and barriers and facilitators of implementation. The results aligned with the five domains of the CFIR. The most cited constructs were the complexity/steep learning curve, cost, and success providers have experienced with neurofeedback. These exploratory findings reveal implementation facilitators and barriers and a starting point for many different research questions exploring possible strategies to increase uptake of neurofeedback in routine mental healthcare.

## Introduction

There are a wide range of clinical tools for the treatment of mental health disorders, but success rates vary depending on treatment type, suitability to the client, and other factors (Barnett et al., 2021). Increasing the use of lesser-known evidence-based treatment modalities may promote treatment use and provide new strategies to improve outcomes. Grounded in the principles of operant conditioning, neurofeedback uses electroencephalogram (EEG)/brain wave data to provide real-time feedback to participants in order to change the functional electrical circuitry of the brain via specialized computer software. In practice, this means that electrodes are used to read the electrical activity of the brains’ surface at specific locations and provide direct and immediate feedback to the brain about its proximity to the activity being rewarded. This feedback can come in many different forms but usually includes both auditory (beeps) and visual feedback (in the form of simplified computer games). Due to this direct, real-time feedback and the adaptivity of the human brain, over time the brain will automatically adjust to the positive changes being encouraged by the feedback and with repetition “learn” this new way of operating.

While originally explored for the treatment of seizures, over the past 60 years neurofeedback has been utilized within a variety of areas of mental health treatment (Evans et al., 2019; Hammond & Novian, 2017). More specifically, neurofeedback has been used as an adjunct to psychotherapy and alternative to medication in the treatment of a variety of diagnoses from attention-deficit hyperactivity disorder (ADHD) to post-traumatic stress disorder (PTSD) (Dupee et al., 2016; Gapen et al., 2016; Kluetsch et al., 2014; Van Der Kolk et al., 2016; Van Doren et al., 2019). Previous research has found neurofeedback to be effective in reducing symptoms of anxiety disorders (Micoulaud-Franchi et al., 2021) and ADHD (Lubar & Shouse, 1976; Van Doren et al., 2019), lowering addiction relapse rates (Saxby & Peniston, 1995; Scott et al., 2005), and reducing affect dysregulation and other symptoms in PTSD (Kluetsch et al., 2014; Lanius et al., 2015; Leem et al., 2021; Micoulaud-Franchi et al., 2021).

However, clinical effectiveness studies are only the beginning of the process of making interventions available to the public (Bauer & Kirchner, 2020; Berenholtz & Pronovost, 2003). Importantly, despite evidence of its effectiveness, neurofeedback is underutilized in routine mental health treatment. Researchers within the implementation science field have provided frameworks and strategies to bridge the gap between intervention effectiveness research and implementation into practice (Bauer & Kirchner, 2020; Eccles & Mittman, 2006), but to date no research has applied implementation science to issues around neurofeedback. The Consolidated Framework for Implementation Research (CFIR) provides an expert consolidation of previous implementation science research models and frameworks into one “meta-theoretical” framework (Damschroder et al., 2009; Damschroder, Reardon, Opra Widerquist, et al., 2022; Damschroder, Reardon, Widerquist, et al., 2022). As stated in the seminal CFIR paper, the framework offers an “overarching typology- a list of constructs to promote theory development and verification about what works where and why across multiple contexts” (Damschroder et al., 2009, p. 2). The CFIR contains five overarching domains that include a variety of factors impacting the implementation of an intervention: *Characteristics of Individuals*, *Inner Setting*, *Outer Setting*, *Characteristics of the Intervention*, and the *Implementation Process* (Damschroder et al., 2009; Damschroder et al., 2022; Damschroder et al., 2022).

To date, the neurofeedback field lacks research focused on determinants and outcomes of implementation (Norris et al., 2024). One study surveyed 71 neurofeedback practitioners and found valuable insights regarding the advantages and disadvantages of neurofeedback, such its effectiveness in symptom reduction and financial issues, but this study lacked a strong foundation in implementation science (Larson et al., 2010). While limited, previous research exploring practitioners’ perspectives on barriers and facilitators to specific mental health interventions outside of the neurofeedback field have provided both practical recommendations to improve access and valuable practitioners’ perspectives regarding these interventions (Baker et al., 2022; Page et al., 2021). For example, Baker found that providers’ a primary perceived barrier of the implementation of cognitive behavioral therapy (CBT) for those living with dementia was a pressure to perform without the adequate support to do so effectively. The CFIR has been utilized across a variety of mental healthcare settings and interventions, such as the use of a complex intervention for dually diagnosed patients with several mental illness and substance use disorders (Rollins et al., 2022). Another study used CFIR to explore the implementation of national clinical guidelines in Sweden for childhood depression and anxiety (Westerlund et al., 2021) found that the guidelines were largely unknown to providers. Another study used CFIR to explore private practice clinicians’ perspectives on determinants of the use of exposure therapy and identified the CFIR domain of individual characteristics to be important (Milgram et al., 2022). Yet another study used CFIR to explore the use of an enhanced implementation support program in community-based substance use treatment finding that providers who were provided extra support utilized the intervention more quickly and more frequently (Helseth et al., 2018). The lack of similar, implementation-focused research in the neurofeedback field represents a large, significant gap. This study will help to fill this gap and inform future neurofeedback implementation research efforts by exploring the perspectives of neurofeedback practitioners’ about determinants of the implementation process. We hope to move the field forward and inform decisions about increasing neurofeedback in routine mental health treatment.

## Methods

### Participants

For this study, we utilized a mixed methods approach with parallel data gathering. This paper will focus on the qualitative portion. The sample of 20 neurofeedback practitioners currently implementing neurofeedback in their outpatient mental health care practices. This study was approved by the University of XXX Internal Review Board (IRB; Protocol #XXXXXX). Participants were recruited in two ways. First, this study was conducted concurrently with a survey study exploring licensed psychotherapists’ interest in and likelihood to implement neurofeedback in their practice. The participants of that study who fit the criteria for the current study (below) were invited to participate in a follow-up qualitative interview. Second, two emails were sent over the span of three months to the EEG Education and Research (EEGer) company mailing list. EEGer is a well-known company based in the United States that develops and sells both the software and training required to practice neurofeedback (www.eeger.com). Participants were required to meet the following inclusion criteria:

- Have active license to practice psychotherapy
- Currently practicing neurofeedback in an outpatient setting
- Have been implementing neurofeedback in their practices for at least 6 months

### Procedures

We conducted semi-structured, one-to-one interviews to gather data on neurofeedback practitioners’ process of being introduced to neurofeedback and beginning to implement the intervention into practice. The interview guide consisted of six broad questions informed by constructs of the CFIR. These interviews were part of a larger mixed methods study. When potential participants expressed interest in participating in an interview, they were sent a link to a demographics survey that included a brief screener. This link was sent via email by the PI or automatically via REDcap, an online survey site used for the surveys mentioned above. This screener included questions that ensured the participant met the inclusion criteria (see Table 1 for more information about screener responses).

Once potential participants completed the REDcap screener, they were contacted by the PI to schedule their research interview. All interviews were conducted via Zoom, audio recorded, and lasted between 20 and 40 minutes. Verbal consent to participate was acquired at the beginning of each interview. All interviews were transcribed verbatim by a transcription service (Otter, https://otter.ai/) and checked for accuracy by the PI (XX). For their participation, interview participants received a discount code for a webinar series provided by EEGer (approximately $75 value).

### Data Analysis

Qualitative data were analyzed using grounded theory analytical techniques —that attempts to recognize the meaning of individuals’ experiences and discover new theories based in real world data (Charmaz, 2014; Glaser & Strauss, 2019). Using grounded theory analytical techniques and informed by the CFIR, the research team created a codebook meant to reflect the process and ideas as they emerged from the data. This coding scheme was developed in an iterative process of discussion and refinement. First, two coders (XX and XX) independently coded three interviews. They independently grouped excerpts of the data into codes and then met to discuss refining the initial codes and group them into categories. The first author (XX) then used the developed codebook to code the remaining interviews, with review and input from a third coder (XX). The codebook was further refined through review of the coded interviews and discussion between the three coders. Once all interviews were coded, the coder most familiar the CFIR (XX) helped connect the broader theoretical categories with the CFIR domains and constructs, and the first author (XX) helped define and describe the central ideas.

## Results

Our sample was mostly White (n = 18, 90%) with an average age of 55 (minimum 34 years, maximum 74 year). Most participants identified as female (n = 15, 75%). Most participants (n = 17) were practicing in the United States. Participants averaged approximately 17.5 years in practice (minimum three years, maximum 36 years) and approximately 8.5 years as a neurofeedback provider (minimum one year, maximum 20 years). Most worked in solo private practices (n = 13, 65%), with three participants (15%) working in group private practice, 2 (10%) in other outpatient settings, and 2 (10%) in community mental health agencies (see Table 1 for more detailed demographic information).

While our initial analysis began with a grounded theory, inductive approach focusing on the two intervention-focused domains of the CFIR, our final codebook aligned well with all five CFIR domains and constructs. Although the interview questions primarily focused on the *Planning*, *Engaging*, *Executing*, and *Reflecting and Evaluating* aspects of the *Implementation Process* and *Implementation Characteristics* as described by the CFIR, the participants also provided other valuable insights into other key facilitators and barriers of implementation across CFIR domains. Results are organized below by the five CFIR domains and subsequent constructs and quotes are provided in Table 2. Throughout this paper, CFIR domains and constructs are italicized for clarity.

### Characteristics of Individuals

Regarding *Knowledge and Beliefs about the Intervention*, some participants described their first impression of neurofeedback and, often, how it changed once they saw the effectiveness of the intervention in their practice. When asked how they first heard about neurofeedback, participants’ responses varied widely from continuing education webinars to their own or loved ones’ personal experiences with neurofeedback. A few participants also spoke to the presence of inaccurate expectations of the implementation of neurofeedback within the field that they learned to navigate early on in their work with neurofeedback. Regarding *Other Personal Attributes*, a few participants talked about burnout, either how their burnout pre-implementation led them to try a new intervention or how using a successful intervention, like neurofeedback, helps stave off burnout. Some participants also mentioned how the personality characteristics of providers likely impact implementation. Specifically, they spoke to the need for providers to enjoy continued learning and have a certain level of comfort with technology. This latter point is important, as using neurofeedback requires technological skill beyond what is usually required of mental health practitioners.

### Inner Setting

Some participants mentioned the importance of individuals within their immediate work setting who helped them with different parts of the implementation of neurofeedback (*Structural Characteristics*). For example, a few participants mentioned administrative staff who helped them with scheduling and billing related to neurofeedback, while others mentioned how supervisors or mentors encouraged their implementation of neurofeedback in a variety of ways. Several participants also described scheduling/time as a barrier to the implementation of neurofeedback for both clients and providers (*Structural Characteristics*). Participants explained that neurofeedback both takes a lot of time on the part of the provider to learn and implement but also can be a scheduling struggle for clients since many practitioners recommend sessions twice per week, which is generally more than conventional psychotherapy models.

### Outer Setting

Almost all participants mentioned some type of continued learning and/or consultation with colleagues and neurofeedback experts as a facilitator to the implementation of neurofeedback in their practice (*Cosmopolitanism*). Most participants mentioned individuals outside of their organization being key to their continued learning via mentoring and community or peer consultation. On the other hand, several participants also mentioned feelings of isolation as a challenge of implementing neurofeedback and their struggle with being the only provider in their area. Several participants mentioned experiences of negativity from other professionals outside of their work setting who were not familiar with neurofeedback but had a negative perception of its implementation.

Some participants mentioned better insurance coverage and/or some type of outside funding as possible facilitators to the implementation of neurofeedback (*External Policies and Incentives*). Several participants described issues within the broader neurofeedback field. For example, they spoke to the neurofeedback field being overly commercialized with industry leaders making claims about their system being superior to others, about the field being “hard to break into,” and the presence of “big egos” within the industry as barriers to its broader implementation.

### Intervention Characteristics

During our interviews, participants often talked about how factors related to the intervention itself—neurofeedback—affected implementation in their practices. They spoke to the need for more research and research funding in order to increase the accessibility of neurofeedback to the public (*Evidence Strength and Quality*). Many participants mentioned the *Complexity* or “steep learning curve” of neurofeedback, and a few specifically mentioned technology as a barrier to implementation. A few participants mentioned the need for better provider education and the need for more help navigating the early stages of implementation (*Design Quality and Packaging*). One participant specifically described feeling overwhelmed when trying to decide which of the myriad of systems to invest in before having a firm grasp of how to successfully implement the intervention.

Many participants described *Cost*—both to providers and to clients—as a barrier to implementation of neurofeedback. Several mentioned that they believe some type of financial support (e.g., grants, insurance coverage, etc.) could increase the accessibility of neurofeedback in routine mental healthcare. One participant mentioned that they were not sure whether they would have ever implemented neurofeedback if they had not been given a scholarship by a foundation that paid for the training and equipment. Another participant mentioned the role a philanthropist played in providing her with equipment and how that facilitated her implementation journey. Several participants mentioned specific ideas about how the help offset the cost barrier for providers, including the use of grant funding, cheaper equipment and training cost, and equipment leasing options. The most common idea of how to help offset cost to clients was insurance coverage, as neurofeedback is currently not covered by most commercial payers in some states or the reimbursement is well below providers’ cash fees. A couple participants reported some fears about insurance companies having oversight over neurofeedback training but still reported that it would be helpful to increase accessibility of neurofeedback. One participant from Canada spoke to how helpful it was for their practice that they could bill for all of their psychotherapeutic services under one broad psychotherapy code, including when they provided neurofeedback during the session.

All participants described either their personal or loved ones’ experiences with neurofeedback and/or successes they have experienced in using neurofeedback with clients as a facilitator to its implementation (*Trialability*). More specifically, most participants talked about how neurofeedback has helped where other interventions fell short in alleviating symptoms in their clients and/or themselves (*Relative Advantage*). Several participants also mentioned how neurofeedback works well alongside other types of care, such as different types of psychotherapy, and some mentioned instances where they have worked alongside other types of providers (*Adaptability*).

### Implementation Process

Almost all the participants talked about how ongoing learning is an essential part of the *Implementation Process* of neurofeedback. Most talked about the important role mentoring had on their implementation, especially in the beginning, and the “steep learning curve” inherent throughout the process of providing neurofeedback (*Planning*). Many participants spoke to the importance of collaborating with their clients during the implementation process and the importance of client education (*Engaging*). Several specifically mentioned the importance of helping clients measure their progress throughout the neurofeedback process (*Reflecting and Evaluating*). Most participants mentioned having started out practicing neurofeedback on themselves and friends and family before bringing it to their practices (*Engaging*). Encouragement from and collaboration with other colleagues was a common theme with many participants as an important ingredient in the implementation process (*Engaging*). Many participants talked about their variety of advertising and referral sources, including networking with other professionals and many sustaining a practice on word-of-mouth referrals (*Engaging*).

## Discussion

The experienced mental health practitioners who participated in this study provided rich data on the determinants of implementing neurofeedback, which could benefit the field of neurofeedback by helping to increase the uptake of the intervention in routine mental health practice. While our interview questions were primarily focused on the practitioners’ individualized process of implementation, interviews revealed several themes that aligned with constructs within all five CFIR domains.

Although several participants described certain personality characteristics of neurofeedback providers (e.g., lifelong learners, comfortable with technology), many participants had significantly varied introductions to neurofeedback and first impressions regarding the intervention. Despite this variation and some of the early barriers mentioned by the participants, all participants found their own way to successfully implementing the intervention. It is important to note that we only interviewed neurofeedback practitioners who had been successfully implementing neurofeedback in their practices for six months or more. Much more could also be learned by interviewing practitioners throughout all/different phases of the implementation process, including those not yet implementing neurofeedback (or even considering it). This could lead to a better understanding of when and why some practitioners either fail to adopt the intervention or give up on the intervention quickly due to barriers.

Most of the participants in this study (n = 13, 65%) practice in solo private practices. Though, we learned about some of the barriers (scheduling/time) and facilitators (inner setting helpers) within the *Inner Setting* domain, these findings are mostly limited to a very specific type of setting. Future neurofeedback implementation research could focus on the differences in the implementation process across settings. This could be especially important given the *Outer Setting* themes that centered around the importance of mentoring and peer/consultation and the barrier that isolation presented for several of the participants.

The complexity of neurofeedback was a common theme throughout our interviews. Most participants mentioned how steep the learning curve is to implement the intervention well. Some even mentioned that without their mentors’ and peers’ support, they might have never started to use neurofeedback or might have given up on using neurofeedback. This finding aligns with Larson et al.’s (2010) theme of a need for “extensive practitioner commitment to overcoming the complexity of neurofeedback.” A few of our participants spoke to the need for more or better provider education early in the implementation process to offset this barrier. Participant recommendations to address lack of adequate provider education on neurofeedback included: a knowledge hub where practitioners interested in neurofeedback can go to learn more about the advantages and disadvantages of different neurofeedback options and systems, instead of information only provided by siloed groups trying to market their own products; introducing neurofeedback in graduate school curriculum to provide an early career introduction to providers; and more collaboration among different types of neurofeedback providers to enhance coherence in the early stages of training. While the complexity of neurofeedback did not ultimately keep our participants from implementing the intervention, it is feasible that this may not be the case for many potential providers. The complexity of neurofeedback should be a focus of future research to help provide insights into how to help providers overcome the learning curve to become successful neurofeedback practitioners.

One of the most significant and expected barriers mentioned in our interviews was financial cost. Similar to the financial issues cited as a disadvantage to neurofeedback in Larson’s findings (2010), participants mentioned both cost to provider and cost to clients as significant barriers. Recommendations to address barriers related to cost for providers include scholarship and/or leasing options to cover training and equipment costs, grant funding, and cheaper equipment development and manufacturing to make the equipment more affordable. The simplest option to address the cost to client barrier would be expanding insurance coverage for the intervention. Future research and advocacy would benefit from exploring why, despite promising efficacy studies, neurofeedback is not a service more commonly covered by health insurance providers and has been prevented from being covered under general psychotherapy codes. The alleviation of this one barrier alone could have an enormous impact on the availability of neurofeedback in routine mental healthcare.

## Conclusion

This study’s exploration of practitioners’ perspectives on the determinants of neurofeedback implementation through the meta-theoretical framework provided by CFIR led to rich insights about the implementation process, the barriers and facilitators to implementation, and possible solutions to address these barriers. The most common themes were the complexity of the intervention and how learning is a key component of the implementation process, cost to both providers and clients as a barrier to implementation, and personal success and/or success with clients as a facilitator of neurofeedback implementation. The field of neurofeedback would significantly benefit from additional in-depth exploration of the specific factors and constructs found in this study.

## Figures and Tables

**Table 1:** Interview participant characteristics (n=20)

Characteristics	N (%)

**Race/Ethnicity**	
White	18 (90)
Mixed race	1 (5)
Other	1 (5)
**Age**	*avg 55.2*
30–39	5 (25)
40–49	1 (5)
50–59	5 (25)
60–69	6 (30)
70+	3 (15)
**Gender identity**	
Female	15 (75)
Male	5 (25)
**Years of practice**	*avg 17.65*
0–5	2 (10)
6–9	2 (10)
10–19	9 (45)
20–29	4 (20)
30–39	3 (15)
**Type of practice setting**	
Community mental health clinic/agency	2 (10)
Solo private practice	13 (65)
Group private practice	3 (15)
Other outpatient setting	2 (10)
**Years practicing neurofeedback**	*avg 8.53*
0–5	8 (40)
6–9	6 (30)
10–19	4 (20)
20+	2 (10)

**Table 2:** Qualitative Analysis Results

CFIR Domain	CFIR Construct	Description/Code	Exemplary Quotation
Characteristics of Individuals	Knowledge & Beliefs a/b the Intervention	- First impression - Inaccurate expectations	*“When I first heard about neuro feedback, it sounded kind of like hocus pocus to me.”*
	Other Personal Attributes	- Burnout - Personality characteristics of providers (i.e., lifelong learners, feelings about technology)	*“I think this is what I will do for my entire career. And I think it saves me from burnout because I feel that I see a lot more progress than with psychotherapy alone.”*
Inner Setting	Structural Characteristics	- Inner setting helpers - Scheduling/Time	*“In an ideal world, if people could come twice a week, that would be great. But for the majority of people who work full time and have children, that’s just not logistically possible.”*
Outer Setting	Cosmopolitanism (networking w/other organizations)	- Continued learning/conferences - Mentoring - Community/Peer consultation - Professional isolation	*“The most important [thing], …besides figuring out a way to afford it, would probably just be having good mentors. Staying connected with other people who are practicing.”*
	External Policies & Incentives	- Insurance coverage - Grants/Outside funding	*“Insurance, get it covered by insurance, and then you’ll have more doctors hear about it, and you’ll have more clients who can do it financially.”*
Intervention Characteristics	Evidence Strength & Quality	- More research & research funding	*“Maybe that has to systemically start at a higher level with more research… which then trickles down to academics, which can then present it to the students who can have an interest in it.”*
	Relative Advantage	- Other inventions fell short	*“You know, when you become a psychotherapist, you’re dealing with people who have really poor regulation. And as a psychotherapist, you can give them all these strategies, but you’re not necessarily changing their regulation. You’re changing their ability to experience the poor regulation…But with neuro feedback, you can change it.”*
	Adaptability	- Neurofeedback alongside other care - Working with other types of care providers	*“And my training of neuro feedback is that it’s not a standalone treatment, it is incorporated into the whole therapeutic process.”*
	Trialability	- Personal experiences with neurofeedback - Success with clients	*“Probably, you know, six, seven sessions into my own work when I realized it was working, and I’d been through many other forms of therapy with many other therapists and hadn’t found the same type of results. I just wanted to be able to provide that for other people.”*
	Complexity	- Technology - Steep learning curve	*“I think I’ve been doing this for five years and I think I’m still learning.”*
	Design Quality & Packaging	- Better provider education - Variety of different systems	*“And then it was the process of trying to figure out what system to use…There’s nowhere to go to find that information…You really just have to be willing to reach out to people and to take a huge leap of faith because you’re putting down a lot of money.”*
	Cost	- Cost/Money/Financial assistance - Grants - Cost to clients - Cost to providers	*“In thinking about why don’t more clinicians use neuro feedback, when it has so much potential, I’d say cost.”*
Implementation Process	Planning	- Learning as part of process - Mentoring - Steep learning curve	*“I think with neuro feedback, you need qualitatively good support… I think those folks that get the equipment and then just kind of stall out there, they might not have found a good mentor, or they just didn’t think it was that important at that point.”*
	Engaging	- Collaboration with clients, client education - Started with self and friends/family - Advertising/Referral sources - Encouragement/Collaboration with colleagues	*“Well, it was having somebody else come to me and say, 'Do you want to do this with me?' And sometimes I wonder, would I have ever done it?”*
	Reflecting & Evaluating	- Client progress measuring	*“And so the I think the most powerful thing about how you implement neurofeedback, is you do it with intention, and towards a clarity of outcome”*
